# First establishment of microsatellite markers in clausiliid snails (Mollusca: Gastropoda: Clausiliidae)

**DOI:** 10.1186/s13104-017-2462-7

**Published:** 2017-03-23

**Authors:** Katharina Jaksch, Luise Kruckenhauser, Elisabeth Haring, Zoltán Fehér

**Affiliations:** 1 Central Research Laboratories, Natural History Museum Vienna, Burgring 7, 1010 Vienna, Austria; 2 Department of Integrative Zoology, University of Vienna, Althanstraße 14, 1090 Vienna, Austria; 3 3. Zoological Department, Natural History Museum Vienna, Burgring 7, 1010 Vienna, Austria; 4 Department of Zoology, Hungarian Natural History Museum, Baross 13, 1088 Budapest, Hungary

**Keywords:** Door snail, Microsatellite, *Montenegrina*, Speciation

## Abstract

**Background:**

Clausiliidae (door snails) are gastropods with a very high diversity concerning shell morphology, especially of their complex closing apparatus, which provides the most important diagnostic traits for classification of taxa. Due to the high variability, a high number of taxa has been described, though their systematics and taxonomy is partially controversially discussed. *Montenegrina* is the second most speciose door snail genus in Europe. It is an obligate rock-dwelling land snail and has, compared to its complex systematics, a rather small distribution range in the western parts of the Balkan Peninsula. The different taxa themselves show a very narrow and patchy distribution range. As *Montenegrina* is comprehensively sampled over the whole distribution range, it is a perfect study system for general questions on speciation and morphological differentiation in land snails. To study the amount of gene flow between geographically close or co-occurring populations, highly polymorphic markers are needed.

**Results:**

Thirteen microsatellite loci with a tetranucleotid repeat were isolated and tested in three geographically close *Montenegrina* populations (two populations of *M. dofleini prespaensis* from the Prespa Lake, n = 35 and one population from *M. stankovici* from the Ohrid Lake, n = 20). The number of alleles per locus ranged from 2 to 27. No significant linkage disequilibria between the same two loci were found in all three tested populations. The deviation from Hardy–Weinberg equilibrium reveal only for two loci a significant deviation from HWE in more than one population (Mont_5483 and Mont_4477).

**Conclusion:**

The 13 newly established genetic markers will help to gain better insights to the population genetic structure of *Montenegrina* and might reveal new results about speciation processes in co-occurring taxa. Furthermore, these microsatellite loci could also be tested in other clausiliid species.

## Findings

The gastropod family Clausiliidae (door snails) is one of the most speciose among land snails and is distributed in four continents with diversity hot-spots in southern Europe, southeastern Asia and the northwestern part of South America [1]. Their eponymous closing apparatus shows several morphological structures (clausilium-plate, lunella-complex, lamellae and plicae), which are very important for discriminating taxa within this family. Due to the high morphological variability and the high number of described taxa, systematics and taxonomy is quite complicated and controversial. Although several clausiliid taxa have been intensively studied concerning phylogenetic [2, 3], phylogeographic [4] and even population genetic questions [5], no microsatellite markers have been applied so far in any of the door snail genera.

*Montenegrina* is an obligate rock-dwelling door-snail genus comprising 106 taxa [6], which are morphologically very diverse. It is the second speciose door snail genus in Europe and has, compared to its complex systematics, a rather small distribution range in the western parts of the Balkan Peninsula. The different taxa show very patchy distribution ranges due to low dispersal ability and the insular occurrence of their calcareous rock habitats. Due to this narrow distribution range and well-known occurrences of many populations, a quite comprehensive sampling is possible, which makes *Montenegrina* a perfect study system for general questions on speciation and morphological diversification of rock-dwelling gastropods. Due to the high morphological variability species delimitation is sometimes challenging in *Montenegrina*, especially in the case of co-occurring morphotypes. Therefore, estimating the amount of gene flow is crucial.

To test for possible gene flow between populations or morphotypes and to get insights into population structure in general, highly polymorphic nuclear genetic markers are needed, which can be applied to a high number of individuals at moderate cost. Therefore, we established 13 microsatellite loci for *Montenegrina* on the basis of next generation sequencing data and tested them in two species, which occur very close together (about 25 km apart). (1) *Montenegrina dofleini prespaensis* Nordsieck, 1988 occurs along the western and southern shores of the Prespa Lake (Albania, Greece). For this study we selected specimen from two populations that live in close vicinity: MprP from Psarades (15 specimens) and MprPE from Panagia Eleousa Cave (20 specimens). (2) *Montenegrina stankovici* (Urbański, 1960) lives along the eastern shore of Lake Ohrid (Macedonia). The studied population MstSV was collected in Sveti Naum (20 specimens).

For DNA extraction we used the Qiagen spin column kit (Qiagen DNeasy Blood & Tissue Kit, Germany) and followed the manufacturer’s protocol. Microsatellite markers were established by ecogenics GmbH (Schlieren, Switzerland) based on seven individuals. Size-selected fragments from genomic DNA were enriched for SSR content by using magnetic streptavidin beads and biotin-labeled GTAT, GATA, AAAC and AAAG repeat oligonucleotides. The SSR-enriched library was analysed on an Illumina MiSeq platform using the Nano 2 × 250 v2 format. After assembly, 13,656 contigs or singlets contained a microsatellite insert with a tetra- or a trinucleotide of at least six repeat units or a dinucleotide of at least ten repeat units. Suitable primer design was possible in 2905 microsatellite candidates, of which 36 were positively tested for functionality and polymorphism. This was done with fluorescent labelled M13 tails that were added to the forward primers [7]. The primers for these 36 microsatellite loci were tested for consistency in PCR amplification and finally the 13 best loci were chosen for further testing in a larger set of individuals. Using the software Multiplex Manager 1.2 [8] these primer sets were then combined in three multiplex PCR reactions (Set1/2/3). For conducting multiplex analyses the forward primers were marked with fluorescent dyes. PCR was performed in 10 µl reaction volume using the Qiagen Multiplex-Kit (1 µl DNA, 2.5 µl Qiagen Multiplex PCR Mastermix, 1 µl primer mix, [1 µl Q-Solution in Set2], 5.5 µl AD [4.5 µl AD in Set2]). The two-step PCR profile started with an initial denaturation at 95 °C for 15 min, two cycles denaturation at 94 °C for 30 s, annealing at 61 °C (Set1) and 58 °C (Set2 and 3) for 90 s, extension at 72 °C for 60 s. For the following 35 cycles the same profile was used, except an altered annealing temperature of 58 °C (Set1) and 51 °C (Set2 and 3), respectively, and a final extension at 60 °C for 30 min. Positive PCR products were diluted 1:10 with AD. 1 µl of the PCR dilution was added to 9 µl of a Hi-Di formamide-Size Standard mixture (0.25 µl, Gene Scan 500 LIZ, Applied Biosystems, USA) and denatured at 94 °C for 4 min. The products were then analysed on a 3130xl Sequence Analyzer (Applied Biosystems, USA). The alleles obtained from the electropherograms were identified and binned using the software GENEMAPPER 5.0 (Applied Biosystems, USA) and checked manually. The multiplex PCR conditions for the 13 loci, the repeat motif and the primer sequences as well as the respective GenBank Accession numbers are given in Table 1.

**Table 1:** 13 Microsatellite loci, primer sequence, repeat motif, primer sets, PCR conditions and accession-numbers

Locus name	Primer sequence (5′–3′)	Repeat motif	Multiplex reaction	Labelling dye	Primer conc.	T_A_	Accession-Nr
Mont_13187	F: TGCCTGCAGTGCGTAGAG	CATA	R1	VIC	2	61/57	KY094088
	R: TATTGATTTCGGACAGGGCG						
Mont_13385	F: GGTAAGCCAATAACGGTGGC	TCTT	R3	PET	2	58/51	KY094089
	R: ATCTGCAACGCCAGTAAACG						
Mont_17419	F: ATAATGTGGGCAGAACAGGC	CAAA	R2	6-FAM	0.5	58/51	KY094090
	R: GTCTTGGGTCGCACAGAATG						
Mont_2349	F: TGACCCGCAGTGATCAAGTC	TTTC	R2	VIC	2	58/51	KY094091
	R: ATCCTATTTCGGTCCCTGGC						
Mont_2916	F: CGTTGAATGAGTCACGGACG	GAAA	R3	VIC	3	58/51	KY094092
	R: ACACTTGTAGGCCGTTGTTC						
Mont_3056	F: GAAAGAAGACCCCATTACGCC	AAAG	R1	6-FAM	1	61/57	KY094093
	R: ATAGCGCACGTCTTTTGTCC						
Mont_3943	F: CGATGATACAAGCAATGCGG	TCTT	R3	NED	2	58/51	KY094094
	R: CCATAGCTGCGTGCTTACAG						
Mont_4042	F: AGCTAAAGTATCGTTGTATGGAGG	TCTT	R1	PET	2	61/57	KY094095
	R: CTCCGAGCATGAGCTTTCTG						
Mont_4196	F: TCAGCCCTGCACAGATAGAC	AAGA	R2	PET	2	58/51	KY094096
	R: ACCTGGACAGCAGTTCTACG						
Mont_4477	F: GTCTGACACAGGCAGCATAC	TTCT	R2	6-FAM	4	58/51	KY094097
	R: CGTTGGTCTCCCGATTTCAC						
Mont_5483	F: ATTCAATGTGCGCAAGTACG	TTTC	R1	NED	3	61/57	KY094098
	R: TGTCATTGATAGCCCCCTCC						
Mont_5717	F: TATGGCCAACGAAACCAAGC	AATC	R3	6-FAM	2	58/51	KY094099
	R: GTTCCGCATGGGACATGATAC						
Mont_5741	F: TGGTATTGAGTGCATAGACGC	GTTT	R3	6-FAM	2	58/51	KY094100
	R: CATTCTGGCGGGGATAAAGC						

*Primer Conc.* primer concentration in pmol; *T*_*A*_ Annealing temperature (°C), 2-step thermoprofile (hot-start T_A_/regular T_A_); *Labelling dye* DS-33 dye set

In total 55 individuals—35 of *M. d. prespaensis* and 20 of *M. stankovici*—were genotyped. For each locus the observed (H_o_) and the expected (H_e_) heterozygosity as well as linkage disequilibria and the deviation from Hardy–Weinberg-equilibrium (HWE) was calculated per population in Genepop 4.2 [9] (Table 2). The number of alleles per locus ranged from 2 to 27. No significant linkage disequilibria between the same two loci were found in all three tested populations, two loci pairs show linkage disequilibrium but only in one population (Mont_2349/Mont_4477 in MprPE; Mont_17419/Mont_5483 in MprP). The deviation from the Hardy–Weinberg equilibrium is significant for four loci in MprP, four in MprPE and three in MstSV. However, checking the results per locus in all populations, reveal only in two cases a significant deviation from HWE over two (Mont_5483) or even all three populations (Mont_4477). The high deficiency of heterozygotes over several loci has already been reported in other gastropod species and could be due to self-fertilization or the patchy distribution leading to drift effects due to restricted gene flow, single loci could be affected by allele scoring artefacts or the presence of null alleles [10]. Thus, the potential presence of null alleles was tested using MICRO-CHECKER 2.2.3 [11]. The results revealed three potential null alleles in the MprP population five in the MprPE population and two in the MstSV population. The loci Mont_4477 and Mont_5483 have potential null alleles in the MprP and in the MprPE population. However, in MstSV these loci give no hint for null alleles and were therefore not excluded from the analysis. At the locus Mont_5717 only one allele per population was found, yet in preliminary tests with other *Montenegrina* species, further alleles are found at this locus. Consequently, the 13 microsatellite markers are suitable for population genetic studies in the rock-dwelling land snail genus *Montenegrina*.

**Table 2:** Population genetic parameters of 13 microsatellite loci in *Montenegrina*

Locus name	N MprP	N MprPE	N MstSV	Size (bp)	Nr. Alleles	He MprP	Ho MprP	HWE MprP	He MprPE	Ho MprPE	HWE MprPE	He MstSV	Ho MstSV	HWE MstSV
Mont_13187	15	20	20	272–448	11	*64.7*	*26.7*	0.000	77.8	80	0.975	–	–	–
Mont_13385	15	20	20	213–227	6	72.9	86.7	0.185	42.4	45	0.771	–	–	–
Mont_17419	15	20	20	181–267	6	28.7	33.3	1.000	*65*	*20*	0.000	–	–	–
Mont_2349	15	20	20	105–253	18	43.4	26.7	0.122	82.4	85	0.701	81.7	85	0.628
Mont_2916	15	20	20	128–272	25	81.1	60	0.003	81.3	85	0.242	*82.7*	*60*	0.286
Mont_3056	15	20	20	178–360	27	64.4	53.3	0.058	*88.8*	*60*	0.004	85.6	85	0.514
Mont_3943	15	20	20	180–444	17	80	73.3	0.069	48.8	45	0.354	68.6	55	0.077
Mont_4042	15	20	20	172–496	21	78.4	86.7	0.735	*79.2*	*55*	0.084	65.8	80	0.751
Mont_4196	15	20	20	133–297	18	81.4	80	0.282	76.9	60	0.244	*70.3*	*30*	0.000
Mont_4477	15	20	20	294–506	23	*84.7*	*40*	0.000	*91.7*	*55*	0.000	9.7	5	0.045
Mont_5483	15	20	20	128–304	17	*52.3*	*20*	0.000	*58.6*	*20*	0.000	53.9	45	0.409
Mont_5717	15	20	20	99–107	2	0	0	n.v.	0	0	n.v.	0	0	n.v.
Mont_5741	15	20	20	193–223	5	48	60	0.577	40.9	45	1.000	23.1	15	0.035

*N* number of individuals per population (Mpr *M. d. prespaensis*. *MprP* from Psarades. *MprPE* from Panagia Eleousa Cave). *Size* (bp) allele size. *H*_*e*_ expected Heterozygosity. *H*_*o*_ observed Heterozygosity. *HWE p* value for deviation from Hardy–Weinberg equilibrium. *n.v*. no value; italic numbers indicate potential null alleles

The newly established genetic markers will be useful in many further investigations within *Montenegrina*. We expect to test gene flow between syntopically occurring congeners, as well as among parapatric and allopatric populations of conspecific taxa. We hope that this will promote the better understanding of speciation patterns and speciation processes in obligate rock-dwelling gastropods. Furthermore, they could also be tested in other clausiliid species.

## Abbreviations

MprP: studied population of *Montenegrina dofleini prespaensis* Nordsieck, 1988, collected in Psarades
MprPE: studied population of *Montenegrina dofleini prespaensis* Nordsieck, 1988, collected in the Panagia Eleousa Cave
MstSV: studied population of *Montenegrina stankovici* (Urbański, 1960), collected in Sveti Naum
H_o_: observed heterozygosity
H_e_: expected heterozygosity
HWE: Hardy–Weinberg-equilibrium
